# Curious Mode of Curing Diseases, Practised by a Tribe of North-American Indians

**Published:** 1814-03

**Authors:** 


*For the Medical and Physical Journal.*

### *Curious Mode of curing Diseases, practised by a Tribe of North-American Indians.*

A METHOD of treating sciatica and chronic rheumatism, by means of exciting perspiration by muscular action, with increased quantity of clothing, was noticed in No. 175, p. 235, of the Medical and Physical Journal. It was suggested to the patient, as Dr. Marcet relates, by a celebrated horse (Vandyk) having been cured of a disorder resembling rheumatism, by sweating in body-cloaths. There is little doubt but this simple remedy may be resorted to in many cases where it is an object to excite perspiration, without injuring the functions of the stomach by the strong medicines usually prescribed on such occasions, under the term of sudorifics. But many cases occur in which the weak condition of the patient would not admit of exercise, with great increase of cloathing. In such circumstances, a practice which obtains among certain tribes of North American Indians, might, perhaps, be practised with advantage by more enlightened people. Father Hennepin, whose book of travels has now become scarce, describes his situation as a prisoner to a savage tribe on the banks of the Mississippi river (or, as he writes, Meschasipi). He was marched during a journey of several days continuance, suffering every species of hardship, till he arrived at the residence of the Indians, when he had the good fortune to be adopted by one of the chiefs. But in consequence of his fatigue and endurance, he was in a very weak and lame condition, and was immediately subjected to the following curious but successful mode of cure.

"This new father of mine observing that I could not well rise without two or three to help me, ordered a stove to be made, which he caused me to enter stark naked with four savages, who, before they began to sweat, tied their *prepuces* about with certain strings made of the bark of a white wood. This stove was covered with the skins of wild bulls, and in it they put flints, and other stones, red hot. They ordered me by signs to hold my breath, time after time, as long as I could, which I did, as well as those that were with me. As for the undecent parts, I had only a handkerchief to cover me. As soon as the savages that were with me had let go their breath, which they did with a great force, *Aquipaguetin* (the Indian chief) began to sing with a loud and thundering voice; the others seconded him; and laying their hands on my body, began to rub it, and at the same time to weep bitterly. I was like to fall into a swoon, and so was forced to quit the stove. At my coming out, I was scarce able to take up my habit of St. Francis to cover me withal, I was so weak. However they continued to make me sweat thrice a week, which at last restored me to my pristine vigor, so that I found myself as well as ever."

If we separate the only useful part of this treatment, viz. the heat and the friction, from that which is superstitious and savage-like, the tying the prepuce, the singing and the weeping, we shall have a rational practice which may be successfully pursued in many cases. I would specify the diseases alluded to in the beginning of this paper, sciatica and chronic rheumatism, stiffness and enlargement of the joints, where acute inflammation was not present. It might also be useful in certain female obstructions, where the constitution is cold and phlegmatic; and in most cases where the warm or vapour baths are adviseable. S. L.

*London, Feb.* 12, 1814.

---

*For the Medical and Physical Journal.*

